# Comparative Study on Local Binary Patterns for Mammographic Density and Risk Scoring †

**DOI:** 10.3390/jimaging5020024

**Published:** 2019-02-01

**Authors:** Minu George, Reyer Zwiggelaar

**Affiliations:** Department of Computer Science, Aberystwyth University, Aberystwyth SY23 3DB, UK

**Keywords:** breast density classification, risk estimation, local binary patterns, texture descriptors

## Abstract

Breast density is considered to be one of the major risk factors in developing breast cancer. High breast density can also affect the accuracy of mammographic abnormality detection due to the breast tissue characteristics and patterns. We reviewed variants of local binary pattern descriptors to classify breast tissue which are widely used as texture descriptors for local feature extraction. In our study, we compared the classification results for the variants of local binary patterns such as classic LBP (Local Binary Pattern), ELBP (Elliptical Local Binary Pattern), Uniform ELBP, LDP (Local Directional Pattern) and M-ELBP (Mean-ELBP). A wider comparison with alternative texture analysis techniques was studied to investigate the potential of LBP variants in density classification. In addition, we investigated the effect on classification when using descriptors for the fibroglandular disk region and the whole breast region. We also studied the effect of the Region-of-Interest (ROI) size and location, the descriptor size, and the choice of classifier. The classification results were evaluated based on the MIAS database using a ten-run ten-fold cross validation approach. The experimental results showed that the Elliptical Local Binary Pattern descriptors and Local Directional Patterns extracted most relevant features for mammographic tissue classification indicating the relevance of directional filters. Similarly, the study showed that classification of features from ROIs of the fibroglandular disk region performed better than classification based on the whole breast region.

## 1. Introduction

It is estimated that one in eight women have the chance of getting breast cancer in their life time [1]. Cancer mortality rates show a slight decline compared to 2012 (15.2/100,000) and the predicted 2020 rate is 13.4/100,000 in Europe [2]. While there is no clear evidence of any single factor which caused the decreasing mortality rate in recent years, the most likely cause has been early diagnosis, treatment and care over the last few decades [2,3,4,5]. Though physical examination is recommended, with this it is difficult to determine breast cancer in its early stages. A variety of medical imaging modalities help in early diagnosis of breast cancer; e.g., mammography, MRI, ultrasound and tomosynthesis. Irrespective of advanced imaging modalities, mammography is still considered as the golden standard for breast screening programs. Mammography provides the radiologist with a visualization of the internal tissue structure of the breast along with information of the amount of glandular and connective tissue relative to the fatty tissue in the breast [6,7,8]. Mammographic density, the relative amount of radiodense tissue, has been considered as a strong risk factor for developing breast cancer (together with gender, age, gene-mutations and family history) [9]. According to many studies, women with high breast density have a higher tendency to develop breast cancer with a two to six-fold increased risk compared to women with low breast density [6,7,10,11,12]. In addition, studies have shown that the sensitivity performance of Computer-Aided Detection (CAD) systems to localize breast abnormalities decreases with increased breast density [13,14]. Glandular tissue in mammographic images is represented as brighter areas while the darker regions represent fatty tissue.

**Figure 1:**
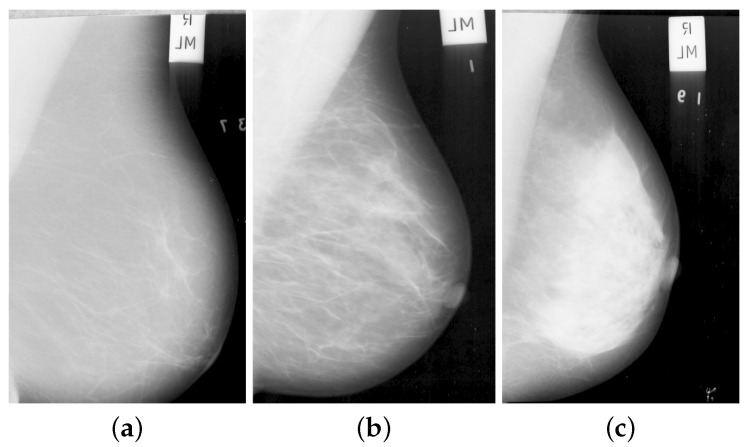
Example mammographic tissue types: (**a**) fatty, (**b**) glandular, and (**c**) dense tissue.

There are different breast classification schemes in the literature based on the breast tissue features used. It has been shown that there is a strong correlation between the breast parenchymal patterns/density and the risk of developing breast cancer and Wolfe et al. classified breast density into five categories [15]. Thereafter, Boyd et al. [16] classified density into six groups based on the proportion of fibroglandular tissue in the breast region. Similar to Wolfe, Tabár et al. [17] classified mammographic images based on the parenchyma rather than on the proportion of breast density. In our study, we used the MIAS dataset, and we classified the mammograms into three categories; Fatty (F), Glandular (G) and Dense (D), based on the tissue structure. As the MIAS dataset provide a three class density classification (F, G, D) as a ground truth and is publically available, we use it in our work. Using three classes helps in controlling the inter and intra- validation variation effect while classifying the mammographic images by different radiologist [18,19]. Example of mammographic images with different tissue type from MIAS dataset are shown in Figure 1. To standardize the mammography density reporting and to minimize the confusion in density interpretations, the American College of Radiology’s Breast Imaging Reporting and Data System (Birads) was developed as a benchmark and quality assurance tool. According to the American College of Radiology (ACR), using the latest (version 5) classification and evaluation of breast tissues used four categories [20]. Similarly, The mammographic density used to be classified into two categories as fatty and dense where the BIRADS I and II considered as fatty while BIRADS III and IV categorized as dense. In order to show the relationship between each category, Muhimmah et al. [21] have performed a comparative study based on mammographic risk assessment.

The classification of mammographic images based on tissue type density and mammographic risk estimation was initiated by Wolfe [15], which was followed by the development of different automatic techniques for breast tissue classification. Developing automatic methods for breast density estimation and classification are appropriate as the sensitivity for detection in mammographic images decreases with the increase in breast density [22]. Most CAD systems have used either segmented breast tissue or a pre-selected breast tissue Region-of-Interest (ROI) for further feature extraction and classification. Diverse features have been used such as histograms [23,24,25], intensity distribution [26], texture based approaches [27,28,29]. While Oliver et al. [6,29] used features based on texture and morphology of tissue patterns, Mustra et al. [30] used GLCM features, Petroudi et al. [12] used textons and statistical features to capture mammographic appearance, Vallez et al. [31] focused on a novel weighted voting tree classification scheme while Bovis et al. [32] used texture features from spatial grey level dependency matrices (SGLD), Fourier power spectrum, Law’s texture measures, and discrete wavelet transform for breast density classification. A detailed study on the datasets and density classification is shown in Table 1 and [33] as the datasets, number of images chosen by researchers are different. With the advent of deep learning in image processing and classification, the technique has been gaining more attention in medical image processing [34,35] worldwide for segmentation and classification processes. Various studies have been performed using deep learning for density classification resulting in promising classification models [36,37,38,39]. But most of the deep learning methods focused on binary classification (fatty or dense) of mammographic breast density.

From the above, it is clear that texture has played a role in density segmentation/classification. Ojala et al. [52] introduced a powerful and computationally simple rotation invariant generic texture classification based on Local Binary Patterns (LBP). LBP and its variants have proven to be useful [29,53] in various medical image analysis applications for extracting local texture features. Zwiggelaar et al. [29] combined Local Binary Pattern (LBP) texture features and texture features extracted from grey level co-occurrence matrices to classify mammograms. Later, Chen et al. [48] performed a comparative study on the performance of LBP, local grey-level appearance (LGA), textons and basic image features obtaining accuracies of 59%, 72%, 75% and 70%, respectively using the whole breast region from the MIAS dataset. We are building on this and we review the use of various LBP variants for density classification.

We have used Elliptical Local Binary Pattern (ELBP) [53], Uniform ELBP, Mean-Elliptical Local Binary Patterns (M-ELBP) [51], Local Binary Pattern (LBP) and LDP (Local Directional Patterns) [54] to capture intrinsic and detailed micro-pattern features from the mammographic images for breast tissue classification into fatty, glandular and dense breast tissue.

In addition to our initial work in [55], the efficiency of M-ELBP texture descriptor was analyzed and compared with other similar LBP variants (LBP, ELBP and Uniform-ELBP). The previous study [55] showed that the texture descriptors like ELBP, U-ELBP and M-ELBP performed better than LBP showing that anisotropic information extracted from breast tissue was potentially more important than a roatation invariant texture descriptor like LBP. A further extension is the inclusion of the Directional texture descriptor LDP [54] is included for analyzing the potentiality of using directionality features of texture descriptors in estimating mammographic breast density.

A further extension to the previous work (where only the ROIs from the fibroglandular region with size equal to 256×256 were considered for classification), this study covered the variation in density classification accuracy while selecting ROIs from different regions of the fibroglandular disk region and is compared to the whole breast.

In addition, the suitable size of ROIs for classification is evaluated by comparing ROIs of different sizes from the fibroglandular disk region. So in summary, the proposed study focused on the difference in the classification accuracy for LBP variants variation in size and location of ROI, selecting whole breast and ROI, the role of machine learning algorithms, the effect of descriptor size, and the role of directional filters in extracting more multidimensional complex texture features for density classification.

## 2. Methodology

Breast ROIs have been used for texture analysis in detecting and classifying different breast abnormalities [56,57]. The analysis and classification of breast density depends upon the breast size and location of the ROI [58,59]. To analyse the effect of ROI location, Li et al. [58] performed a risk estimation on breast parenchymal patterns on breast ROIs at different locations behind the central nipple area using texture features. The study showed a significant decrease in performance when the ROI location moved backwards from the central region. Similarly, Sharma et al. [59] completed a study to analyze the effect of the size of the ROIs for classification and concluded that larger ROIs (above 200×200 pixels) did decrease the classification accuracy due to inclusion of more tissue and smaller ROIs were affected by lose of important tissue pattern features.

### 2.1. Pre-Processing

**Figure 2:**
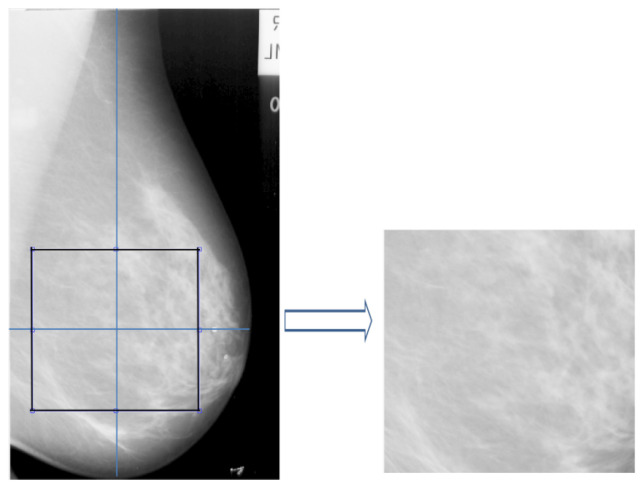
ROI extraction from the fibro-glandular disk region.

The mammographic images are pre-processed to remove the pectoral muscle and other artifacts (e.g., labels; see Figure 1) to extract the main breast region for processing [60]. The method, developed by the Chen and Zwiggelaar [60] separated the background region containing the annotations, labels and frames from the whole breast as the initial step. Subsequently, the method used histogram thresholding, contour growing and polynomial fitting method to remove the pectoral muscle from the breast tissue region. As most of the dense tissue and parenchymal patterns are situated within the breast fibroglandular disk area (the central region behind the nipple), we extracted ROIs from that region. The ROI was extracted ([51] for details) from each image in the MIAS database [61] with size equal to 256×256 pixels as shown in Figure 2. The longest vertical and horizontal distance within the breast was estimated and the intersection point of the both lines was considered as the central point of the fibroglandular region for extracting the ROIs (see Figure 2). With this intersection point as the center, the ROIs of size 256×256 were extracted. Noise reduction was performed on the extracted ROI using a median filter of 3×3 size.

### 2.2. Feature Extraction and Feature Selection

**Figure 3:**
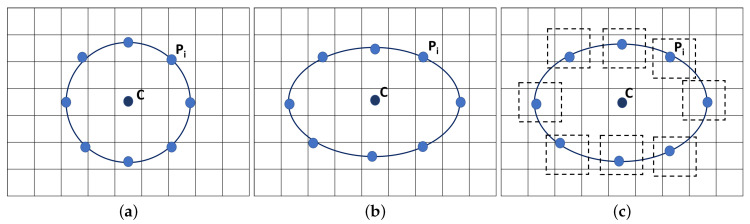
LBP pattern for (**a**) $LBP^{P,R=2}\left(x_{c},y_{c}\right)$, (**b**) $ELBP^{P,R_{1}=3,R_{2}=2}\left(x_{c},y_{c}\right)$, (**c**) M-ELBPP, $R_{1}=3,R_{2}=2$ ($x_{c},y_{c}$) where *P* = 8.

Ojala et al. [52] proposed a local texture descriptor, Local Binary Pattern (LBP) for texture feature extraction. Besides the simplicity and robustness to variation in image intensity levels, its computational efficiency offers a wide range of application in texture feature extraction. In the classical LBP developed by Ojala et al., for each central pixel $\left(x_{c},y_{c}\right)$ in the input image with a grey level value $g_{c}$, its LBP value is estimated by comparing the $g_{c}$ value with the grey level values of pixels at *R* distance within its surrounding *P* neighbourhood pixels following the pixels along a circular path either clockwise or counter-clockwise (see in Figure 3a). If the central pixel value $\left(x_{c},y_{c}\right)$ has a higher grey level value than the neighbouring pixel $P_{i}$, the neighbour pixel is assigned a value 0 else 1 giving a *P*-bit binary number. Using ROI level histogram information provides robustness, although it removes localisation, which prevents detailed segmentation information. Although this can be addressed by using small local ROIs/windows, but this is seen as future work (the histogram of LBP values for the whole ROI was calculated). For a neighbourhood of eight, LBP can generate 256 different binary patterns. The $LBP^{P,R}$ is calculated as follows:

(1) $$LBP^{P,R}\left(x_{c},y_{c}\right)=\sum_{i=1}^{P}s\left(g_{i}^{P,R}-g_{c}\right)2^{i-1}$$

where s(x) is defined as

(2) $$s\left(x\right)=\left\{\begin{array}{cc} 1, & \mathrm{if}\;x>=0\\ 0, & \mathrm{if}\;x<0 \end{array}\right.$$

A circular neighbourhood is an advantage for a texture descriptor but there are applications were the anisotropic texture information have more potential in distinguishing objects. A circular-like topologies like an ellipse helps in attaining features from different directions [62]. For Elliptical Local Binary Patterns (*ELBP*), for each central pixel $\left(x_{c},y_{c}\right)$, we consider the neighbouring pixels *P* which lie on an ellipse (with radius $R_{1}$ and $R_{2}$) which was calculated as:

(3) $$ELBP^{P,R_{1},R_{2}}\left(x_{c},y_{c}\right)=\sum_{i=1}^{P}s\left(g_{i}^{P,R_{1},R_{2}}-g_{c}\right)2^{i-1}$$

where the *i*th neighbouring pixel coordinate of $\left(x_{c},y_{c}\right)$, is computed as:

(4) $$angle_{step}=2*\frac{\pi }{P}$$

(5) $$x_{i}=x_{c}+R_{1}*\cos\left(\left(i-1\right)*angle\;step\right)$$

(6) $$y_{i}=y_{c}-R_{2}*\sin\left(\left(i-1\right)*angle\;step\right)$$

Compared to the LBP patterns, the ELBP descriptors help in extracting more specific spatial features from the mammographic images as it could extract additional directional features at different orientations covering additional micro patterns. For the computation of the Mean-Elliptical Local Binary Patterns (*M-ELBP*), we consider the mean intensity values (${\bar{g}}_{i}$) around each neighbouring pixel $P_{i}$ which lie on an ellipse (with radius $R_{1}$ and $R_{2}$) as

(7) $${\text{M-ELBP}}^{P,R_{1},R_{2}}\left(x_{c},y_{c}\right)=\sum_{i=1}^{P}s\left({{\bar{g}}_{i}}^{P,R_{1},R_{2}}-g_{c}\right)2^{i-1}$$

The mean intensity value around each neighborhood pixels (a local window of size 3×3 was used here) is compared with the central pixel to create a binary pattern as in LBP or ELBP.

**Figure 4:**
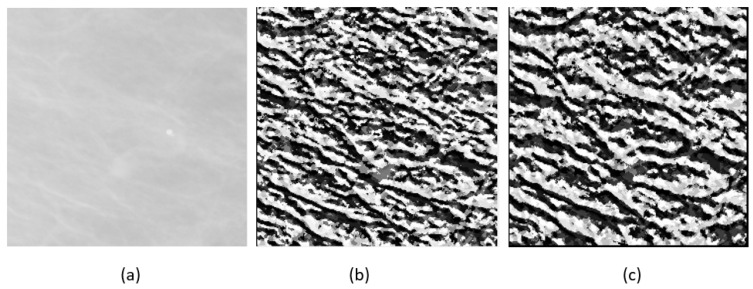
Effect of horizontal and vertical *M-ELBP* on mammographic RoI (**a**) Original Image (**b**) *M-ELBP* pattern for *M-ELBP*${}^{8,7,4}$ at $\theta =0^{\circ }$ (**c**) *M-ELBP* pattern for *M-ELBP*${}^{8,4,7}$ $\theta =90^{\circ }$.M ELBP PATTERNS

**Figure 5:**
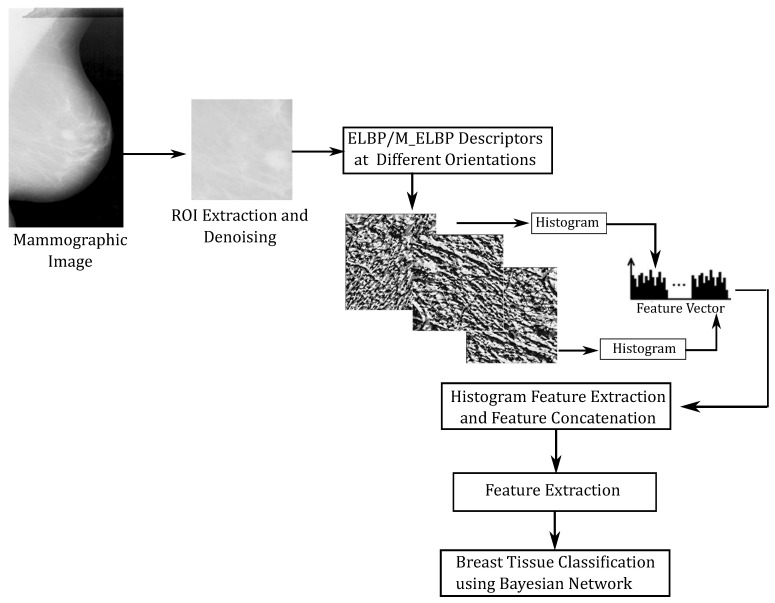
Summary of ROI selection, feature extraction and classification using ELBP variants.

We used the ELBP/M-ELBP descriptors at eight different orientations generating an eight-bit pattern for each central pixel. The experimental results by [63], indicated that extracting tissue features from multiple orientations performed better in determining the mammographic tissue class due to the complex and multidimensional appearance of tissue patterns in the breast. When $\left(R_{1}=R_{2}\right)$, the ELBP/M-ELBP reduces to the LBP descriptor. To extract additional features, we modified the ELBP operator to the mean-ELBP (M-ELBP) where the intensity features at different orientations were attained along with the texture features [51]. A detailed overview of the various method is described in Figure 3. Compared to the LBP descriptor, the elliptical descriptors could extract more features when applied to ROIs at different orientations $\theta =\{0^{\circ },45^{\circ },90^{\circ },135^{\circ },180^{\circ },225^{\circ },270^{\circ }$ and $315^{\circ }\}$. Figure 4 shows the effect of horizontal and vertical M-ELBP on the extracted image ROI for *P*=8 at an orientation $\theta =0^{\circ }$ and $\theta =90^{\circ }$. The procedure is repeated for all orientations to extract M-ELBP pattern features. Figure 5 summarizes the classification of breast tissue using the ELBP/M-ELBP descriptor variants at eight different orientations with an elliptical radius of ($R_{1}=4,R_{2}=7$) for a neighbourhood size of eight pixel. Subsequently, the histogram labels for the ELBP/M-ELBP descriptors at all orientations are concatenated to generate the texture feature vector for the ROIs.

A useful modification to the LBP operator was the introduction of the uniform patterns [64] concept to the LBP which can reduce the length of the feature vector from 256 to 59 for an 8- bit binary pattern. The uniform pattern concept was developed from the fact that certain binary patterns occur commonly in texture images. If a binary pattern contains at most two transitions of 0->1 or 1->0, the pattern is called uniform. So, while constructing histogram, the uniform patterns have separate bins with a single bin assigned to all other non-uniform patterns.

Due to the feature extraction at multiple orientations, the feature vector length when concatenated will be large (each orientation for the ELBP descriptor generated 256 size histogram) and the probability of correlation between features increases. To retain only prominent features and to reduce the computational cost due to high dimensionality, feature selection was performed on the extracted feature vector. A correlation based feature subset (CFS) selection method [65] with a best first search method was used. Highly correlated feature subsets that are highly correlated with the group class while having lower inter correlation among feature subsets are preferred for attribute selection. This calculated the individual predictive ability of each attribute/feature in the dataset along with the redundancy between each feature. The most prominent selected features were then fed into various classifiers for classification of mammographic breast tissue.

Additionally, to compare the directional feature extraction capability of the M-ELBP descriptor and the importance of directional texture feature descriptors for extracting mammographic parenchymal features, we compute the classification accuracy of the Local Directional Pattern (LDP) [66]. The LDP produces an eight-bit binary pattern similar to LBP for a neighbourhood of eight. Unlike LBP, the LDP patterns are computed by comparing the relative edge response values of each pixel at different orientations. In order to get the edge responses, the eight directional edge response values of a particular pixel were calculated using Kirsch masks ($(M_{0}-M_{7}$, also known as the Kirsch compass kernel which is a non-linear edge detector to find the maximum edge strength in eight predetermined compass directions with 45 degree increments). The edge responses are not equally important in all directions ($m_{0},m_{1},...,m_{7}$) as the corners and edges show high responses for particular directions. So the top *k* values from $|m_{j}|$ were selected and defined as ’1’ while the other (8−k) bits were set to ’0’.

### 2.3. Extraction of ROIs

Subsequently, to study the effect of classification using the whole breast and different sized ROIs, varying sizes of ROIs were extracted ranging from 256×256 pixels, 200×200 pixels and 100×100 pixels. All the ROIs were selected randomly from the 256×256 pixels ROI region (used for descriptor comparison) selected from the fibroglandular disk region.

Similarly, to study the effect of the descriptor size on tissue classification, we performed the experiment as described in Figure 5 with varying size of $R_{1}$ and $R_{2}$ ($R_{1}=\left\{2,4,6,12\right\}$ and $R_{2}=\left\{5,7,9,15\right\}$). For each elliptical size (varying sizes of $R_{1},R_{2}$) with a neighborhood of eight, the feature extraction is done for eight different orientations. The extracted features at different orientations are concatenated and feature selection (extracting the most prominent features) is done as in Figure 5. Tahmassebi et al. [67] showed the importance of ranking and selecting the best extracted parameters while considering medical image data.

## 3. Experimental Results

The evaluation presented in this paper was performed on the Mammographic Image Analysis Society (MIAS) dataset [61]. The database contains 322 mammographic images of 161 patients with mediolateral-oblique views. Each mammogram is classified into three distinct groups as either Fatty (F), Fatty-Glandular(G) or Dense-Glandular (D) based on the tissue pattern. We perform classification on 321 images (one image was not available for historical reasons) with 106 fatty cases, 104 fatty-glandular and 111 dense-glandular cases.

### 3.1. Comparison between LBP Variants

For the comparison of classification by different Local Binary Pattern variants, we performed LBP, ELBP, uniform-ELBP and M-ELBP and LDP on ROIs of size 256×256 from the fibroglandular region with neighbourhood size equal to eight. While LBP extracted features only from a circular neighbourhood, the ELBP was able to extract more structural and spatial features at different orientations extracting multidimensional micro-pattern features of the breast tissue. In-order to incorporate the intensity features along with the textural features into the histogram, we used M-ELBP. Similarly, the effect of Uniform patterns in tissue classification was estimated using Uniform ELBP [68]. To keep the consistency of classification accuracy due to the classifier effect, we used the Bayesian Network for every descriptor with 10-fold cross validation.

Table 2 shows the classification results on the ROIs from the MIAS database using LBP. The approach gave a classification accuracy equal to 70.0%. Since the LBP operator considered a circular pattern of the neighbourhood, it could not capture the directional features of the breast tissue due to its rotation invariant feature.

Table 3 shows that the classification accuracy for the ELBP descriptor has improved to 75.0% compared to LBP showing that ELBP can perform better by extracting additional multidimensional features from different directions. In-order to study the effect of uniform patterns in the classification of density, we performed Uniform-ELBP and found results similar to ELBP giving an accuracy of 74.0% as shown in Table 4.

To understand the effect of intensity features in mammographic breast tissue classification, our proposed variant of ELBP [51], M-ELBP was used which classified the mammographic images from the MIAS dataset into BIRADS classes obtaining a classification accuracy of 74%. Table 5 shows that the classification accuracy for this ELBP variant (M-ELBP) improved to 79.8% while including intensity features to texture pattern in breast tissue classification.

Similarly, to analyze the role of directional texture descriptor for mammographic density estimation, we used the LDP [54] operator on the same set of ROIs with the Bayesian Network. The classification results in Table 6 show an accuracy of 75.4%, which was lower than the M-ELBP descriptor for the Bayesian Network classifier but shows the strength of pixel directional response in feature extraction.

When considering Table 2, Table 3, Table 4, Table 5 and Table 6, it seems there is a trend for over-estimation, which could be due to the wide variation in glandular tissue appearance within the dataset and the ROI selection process. For the classification results for M-ELBP (see Table 5) it should be noted that this is the only approach which does not have any cases which are mis-classified by more than one class (so no Fatty cases were classified as Dense and visa versa), which could be caused by the incorporation of texture features along with the mean intensity values of the neighbourhood making it more robust to the tissue pattern and intensity variations. In addition, it should be noted that the LBP based approach performs worse for the Dense class (which is probably caused by the rotational invariant aspects), whilst both ELBP and uniform-ELBP perform less on Glandular cases (which could again be caused by the wide range of variation in the Glandular class and the effects of noise). We see a more detailed analysis of these aspects as future work, which could be based on an in-dept analysis of the feature space.

In order to analyze the significance of the test for different texture descriptors [69], we perform a ten-run ten-fold cross validation for all the texture descriptors. Table 7 shows that the Bayesian Network yielded an accuracy of 69.44±0.92, 75.41±1.05, 72.86±1.06, 77.38±1.06 and 74.92±0.67 for LBP, ELBP, U-ELBP, M-ELBP and LDP, respectively. Table 7 also indicates if differences were significant (at the 0.05 level).

### 3.2. Study on Classifier Effect on Classifications

Machine learning approaches have a great impact on medical image classification and can provide valuable predictive information to guide treatment decisions [70]. In order to understand the classifier effect on mammographic density estimation accuracy, we computed the classification using different classifiers for the same feature set. The classification accuracy illustrated by Table 7 shows the variation in classification accuracy for a 10 run 10 fold cross validation for different classifiers showing the importance of feature selection and classifier effect on density classification. The classifiers chosen for our experimental study are Bayesian Network, K-Nearest neighbor classifier, Support Vector Machine, and Random Forest [71,72]. Additionally, to test the statistical significance of classification accuracy, we have performed a unpaired t-test for a significance level of p=0.05 between descriptors and classifiers (see in Table 7) taking best classification result by the respective classifier for each descriptor as base line.

**Figure 6:**
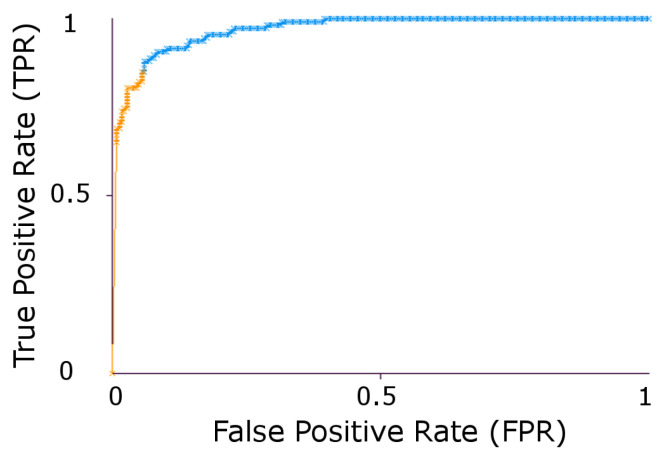
ROC curve for M-ELBP for a Bayesian Classifier.

Similarly, the area under the ROC curve ($A_{z}$) [73] was calculated to compare the efficiency of classifiers for density estimation. It represents how good a classifier can distinguish between different groups. Table 8 shows results using stratified 10 run 10 fold cross validation for $A_{z}$. On comparing the classification accuracy and $A_{z}$ results from Table 7 and Table 8, LDP and M-ELBP show a balanced result for classification accuracy by same classifiers showing the potential of directional filters for mammogram density classification. Similarly, Figure 6 represents the ROC curve for M-ELBP descriptor using the Bayesian classifier.

From the experimental studies, the M-ELBP and LDP performed better in classifying the mammographic breast tissue showing the importance of directional filters in feature extraction. In-order to avoid the classifier effect on classification accuracy, we use the Bayesian network for further experiments in the paper.

### 3.3. Study on Descriptor Size

**Figure 7:**
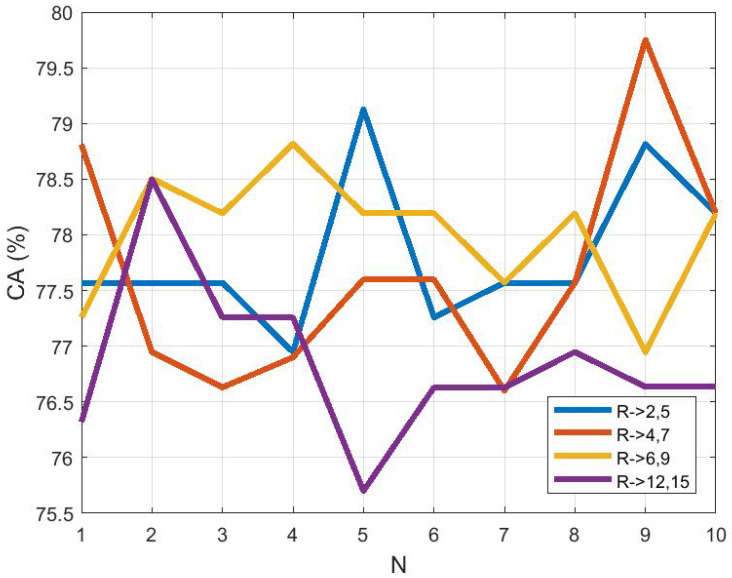
Classification accuracy as a function of descriptor size (N indicates a distinct run).

**Figure 8:**
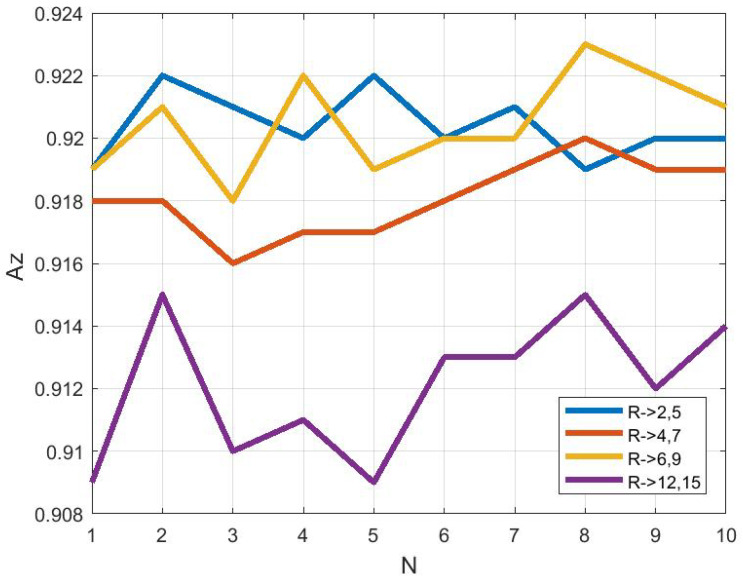
Area under ROC curve $A_{z}$ as a function of descriptor size (N indicates a distinct run).

To compare the effect of descriptor size on classification, the M-ELBP operator with varying size of R1={2,4,6,12} and R2={5,7,9,15} were applied for the ROI size equal to 256×256 pixels. In order to compute the stability of the approach we used a 10 run 10 fold cross validation scheme using a Bayesian network. The results showed that the descriptor classification remained stable with the change in size. Figure 7 shows the classification accuracy (CA%) to be 77.82±0.68, 77.66±1.01, 78.01±0.57 and 76.85±0.73. The figure represents the classification accuracy obtained by the Bayesian Network classifier for each run (*N* = number of runs) for a 10-fold cross validation scheme. Similarly, from Figure 8, the area under the ROC curve($A_{z}$) shows similar results as 0.9204±0.0010, 0.9181±0.0011, 0.9205±0.0015 and 0.9121±0.0022 for the M-ELBP descriptor of size R1={2,4,7,12}, R2={5,7,9,15} with a neighbourhood of eight pixels, respectively.

### 3.4. Effect of ROI Size and Location in Classification

**Figure 9:**
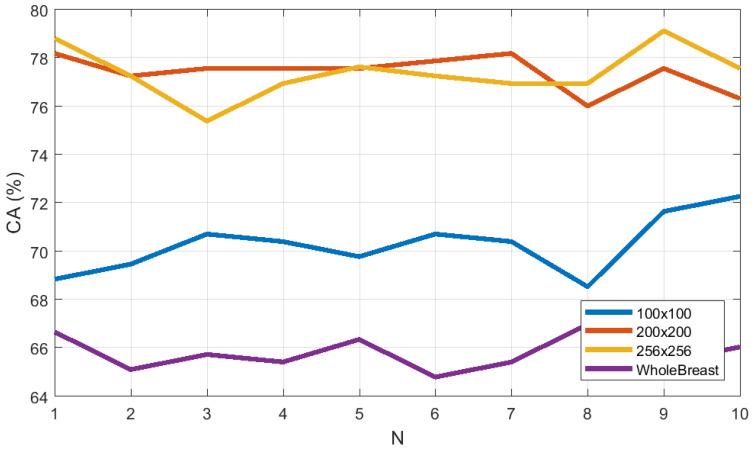
Classification accuracy as a function of ROI size (N indicates a distinct run).

**Figure 10:**
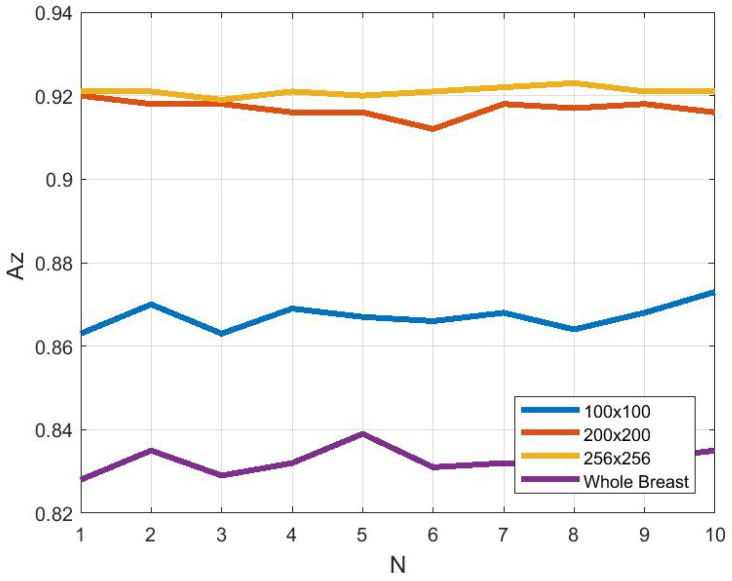
Area under ROC curve ($A_{z}$) as a function of ROI size (N indicates a distinct run).

Further experiments were done to estimate the effect of the ROI size and location in determining the classification accuracy. We estimate the classification accuracy while using the whole breast, or ROI sizes varying from 100×100, 200×200 to 256×256 pixels were selected from the fibroglandular disk region. The classification results for the M-ELBP descriptor with size R={4,7} and P=8 using the Bayesian Network classifier for 10 run 10- fold cross validation. Figure 9 and Figure 10 shows the variation in classification accuracy (*N* indicate a distinct run) while the whole breast region (CA%=65.79±0.70, $A_{z}=0.83\pm 0.003$) or an ROI of size 100×100 pixels (CA%=70.28±1.16, $A_{z}=0.86\pm 0.003$) are selected. The most appropriate ROI size for this study was found to be between 200×200 pixels (CA%=77.41±0.72, $A_{z}=0.91\pm 0.002$) to 256×256 pixels (CA%=77.39±1.04, $A_{z}=0.92\pm 0.001$) from the fibroglandular region due to the presence of the appropriate parenchymal patterns. This showed that if the ROI size is small, classification can be affected due to minimum dominant features. Similarly, if the ROI size is too large or include irrelevant regions like tissue near the pectoral muscle or the breast boundary regions can result in extracting less discriminant features. In case of choosing the whole breast, the classification can be affected by irrelevant data resulting in a poor classification accuracy as it extracted multi-class texture information making it difficult for discriminating the prominent features for each density class. So the study showed that choosing the correct ROI size and location can lead to improved density classification inline with similar studies in the field [58,59].

## 4. Discussions and Conclusions

Due to variations in datasets, classifiers, number of images used by researchers, difference in density classes a direct comparison of our results with existing methods is difficult. So the state of art with the same dataset and density classes are considered for comparison with our studies. Muhimmah et al. [23] classified breast density into 3 classes using multi resolution histogram information using an SVM classifier obtaining an accuracy of 77.57%. Similarly, Subashini et al. [43] used 43 images from the MIAS dataset using a segmented breast tissue approach and attained an accuracy of 95.55% with a SVM classifier using statistical features. But the good classification results could be due to the limited number of images selected and so the classification results would probably decrease with whole database. Blot et al. [41] used background texture information for 265 images and obtained a CA of 65.0% using a KNN classifier. Zwiggelaar et al. [24] used gray level histogram features for three class density classification on MIAS dataset with a subset of 312 images and attained an accuracy of 71.5%. Compared to the methods used for breast density classification, the Local Binary Pattern variants such as traditional LBP, ELBP, U-ELBP, M-ELBP, LDP obtained CA value of 69.44±0.92, 75.41±1.05, 73.29±0.64, 77.38±1.06, 75.95±0.96 respectively (see Table 7). Comparison with the results in Table 1 shows that the Local Binary Pattern variants are competitive in extracting relevant features for breast density classification. Thus our results are comparable with the state of art while considering the number of images (321) and the density class used for classification.

It is evident from the Table 7 and Table 8 that though circular neighborhood provide rotation invariance as in the traditional LBP, elliptical topologies could extract more anisotropic features from an image especially for images which do not have a specific pattern like mammogram tissue which are complex in nature. So here, the elliptical texture descriptors performed better compared to traditional LBP information. Additionally, elliptical descriptors performed better compared to traditional LBP variants as it extract more complex texture patterns due to its directionality nature along with the possibility of multi-scale texture analysis. Similarly, the results showed that incorporating image intensity features like the mean with texture features (as in M-ELBP) improved the feature vector strength in distinguishing different patterns for a complex image. Similarly, results from the LDP descriptor showed the potentiality of directional filters and importance of analyzing the strength of edge and corner features of pixels in an image in classifying patterns. Unlike traditional LBP, both M-ELBP and LDP is better dealing with noise in the image as it is not directly comparing the gray values (contrast) between pixels. Therefore it helped in extracting the important parameters for classification.

Additionally, this study concentrated on analysing the effect of selecting the region of interest (ROI) from mammographic images for breast tissue classification. The study showed that selecting the size of the ROIs at 200×200 pixels and 256×256 pixels taken from fibroglandular disk region were most appropriate. This showed similar result to the study performed by Li et al. [58] where ROIs were selected at different distances from the breast nipple. A similar study was performed by Rampun et al. [63] showing the importance of choosing the ROIs from the fibroglandular region for density classification.

Further results on the selection of the ROI size showed that smaller ROIs can lead to decreased classification due to lack of texture patterns extracted and very large ROIs can include irrelevant features, again resulting in decreased classification. The ROI size indicated is comparable with the results shown by Sharma et al. [59].

For future work, we will focus on developing a method which extracts additional multidimensional directional pattern features by focusing on the edge responses of each pixel and gradient changes. Additionally, the classification accuracy of boosting algorithms like XGBoost based on gradient boosting will be analyzed [74]. Similarly, combining handcrafted features focusing on pattern responses with deep learning approaches for feature extraction and classification can be an advancement in the field of breast density classification. Although several studies [36,37,38,39] have focused on deep learning features for mammographic density classification, a comparison of handcrafted features (focusing on intensity and local patterns) to deep learning feature extraction and classification is a potential area of investigation.

## Figures and Tables

**Table 1:** A summary of existing approaches on breast density classification indicating authors, years of publication, techniques, experimental data for test, and classification.

Author	Features	Classifiers	Dataset	Number of Images	Result	Density Class	Year
Karssemeijer [40]	Grey level histograms	KNN	Nijmegen	615	65.0%	BIRADS I-IV	1998
Blot and Zwiggelaar [41]	Texture GLCM	KNN	MIAS	265	65.00%	Fatty, Glandular and Dense	2001
Bovis and Singh [32]	SGLD	ANN	DDSM	377	71.40%	BIRADS I-IV	2002
Petroudi et al. [42]	Textons based on MR8 filters	KNN	Oxford	132	75.75%	2 Class-Fatty and Dense	2003
Zwiggelaar et al. [24]	Gray level histogram	PCA +KNN	MIAS	312	71.5%	Fatty, Glandular and Dense	2005
Oliver et al. [29]	Morphological Features	KNN Decision Tree	MIAS DDSM	270 300 R-MLO	67.0% 73.0% 47.0%	BIRADS I-IV	2005
Muhimmah and Zwiggelaar [23]	Multiresolution histogram Features	DAG-SVM	MIAS	321	77.57%	Fatty, Glandular and Dense	2006
Oliver et al. [6]	Texture	KNN+SFS (sequential forward selection (SFS))	MIAS DDSM	322 831	86.0% 77.0%	BIRADS I-IV	2008
Subashini et al. [43]	Statistical features	SVM	MIAS	43	95.55%	Fatty, Glandular and Dense	2010
Oliver et al. [44]	Connected density clusters taking the spatial information	LDA-PCA	DDSM	831	79.0%	Fatty and dense	2010
Zwiggelaar [45]	LGA	KNN	MIAS	322	64.0%	BIRADS I-IV	2010
Liu et al. [46]	Statistical	SVM	Tianjin Tumor Hospital	88	86.40%	BIRADS I-IV	2011
Tzikopoulos et al. [47]	Intensity based	SVM	MIAS	322	85.70%	Fatty, Glandular and Dense	2011
Chen et al. [48]	LBP LGA BIF Textons Topographic	KNN, Bayesian	MIAS	322	59.0% 72.0% 70.0% 75.0% 76.0%	BIRADS I-IV	2011
Qu et al. [49]	Unknown	E-FELM (Evolutionary Fuzzy Extreme Learning Machine)	MIAS	322	72.6%	BIRADS I-IV	2011
Muštra et al. [30]	GLCM	KNN+Naïve Bayesian	KBD-FER, University Hospital Dubrava, Zagreb	144	79.30%	2 Class-Fatty and Dense	2012
Muštra et al. [30]	GLCM	KNN+Naive Bayesian	MIAS	144	82.0%	2 class-Fatty, Dense	2012
He et al. [28]	Texture	Binary model based Bayes Classifier	MIAS	322	78.0%	BIRADS I-IV	2012
Rampun et al. [50]	LTP	SVM	MIAS	322	82.3%	BIRADS I-IV	2017
George et al. [51]	M-ELBP LBP	Bayesian	MIAS	321	74.0% 66.5%	BIRADS I-IV	2018

**Table 2:** Confusion matrix for automatic tissue classification using Local Binary Pattern (LBP) descriptor for ROI size 256×256.

	Automatic Classification		
Truth Data	**Fatty**	**Glandular**	**Dense**
**Fatty**	86	19	1
**Glandular**	16	73	15
**Dense**	7	38	66

**Table 3:** Confusion matrix for automatic tissue classification using the Elliptical Local Binary Pattern (ELBP) descriptor for ROI size 256×256 and R={4,7} and P=8.

	Automatic Classification		
Truth Data	**Fatty**	**Glandular**	**Dense**
**Fatty**	86	20	0
**Glandular**	11	68	25
**Dense**	3	22	86

**Table 4:** Confusion matrix for automatic tissue classification using the Uniform-Elliptical Local Binary Pattern (uniform-ELBP) descriptor for ROI size 256×256 and R={4,7} and P=8.

	Automatic Classification		
Truth Data	**Fatty**	**Glandular**	**Dense**
**Fatty**	86	19	1
**Glandular**	11	69	24
**Dense**	2	27	82

**Table 5:** Confusion matrix for automatic tissue classification using the Mean Elliptical Local Binary Pattern (M-ELBP) descriptor for ROI size 256×256 and R={4,7} and P=8.

	Automatic Classification		
Truth Data	**Fatty**	**Glandular**	**Dense**
**Fatty**	92	14	0
**Glandular**	13	76	15
**Dense**	0	23	88

**Table 6:** Confusion matrix for automatic tissue classification using the Local Directional Pattern (LDP) descriptor for ROI size 256×256.

	Automatic Classification		
Truth Data	**Fatty**	**Glandular**	**Dense**
**Fatty**	92	13	1
**Glandular**	10	74	20
**Dense**	3	32	76

**Table 7:** Classification accuracy results by various classifiers for LBP variants for ROI size 256×256 pixels for 10-run 10-fold cross validation. (* indicates that the CA% are not significantly different from the best result using an unpaired t test (p=0.05) level).

Classifier	LBP	ELBP	Uniform ELBP	M-ELBP	LDP
**Bayesian Network**	69.44±0.92	75.41±1.05	72.86±1.06	77.38±1.06	74.92±0.67
**KNN**	69.34±1.09	70.43±1.03	71.46±1.41	$75.46\pm 0.78\;{}^{*}$	75.95±0.96
**SVM**	67.07±0.75	73.11±0.76	73.29±0.64	74.42±0.92	$74.17\pm 0.52\;{}^{*}$
**Random Forest**	69.19±1.31	73.26±1.08	73.04±0.97	73.20±1.36	75.91±1.01

**Table 8:** Area under ROC ($A_{z}$) classification results for LBP variants using ROI size 256×256 pixels for a 10 run 10 fold cross validation. * indicates that the CA% are not significantly different from the best result using an unpaired t test (p=0.05) level).

Classifier	LBP	ELBP	Uniform ELBP	M-ELBP	LDP
**Bayesian Network**	0.89±0.006	0.90±0.001	0.89±0.003	0.92±0.000	0.89±0.004
**KNN**	0.86±0.005	0.85±0.003	0.87±0.005	$0.89\pm 0.005\;{}^{*}$	0.89±0.002
**SVM**	0.78±0.004	0.83±0.005	0.84±0.003	0.85±0.005	$0.84\pm 0.002\;{}^{*}$
**Random Forest**	0.86±0.004	0.89±0.004	0.89±0.004	$0.89\pm 0.004\;{}^{*}$	0.90±0.003
